# Sex, Prescribing Practices and Guideline Recommended, Blood Pressure, and LDL Cholesterol Targets at Baseline in the BARI 2D Trial

**DOI:** 10.1155/2015/610239

**Published:** 2015-03-19

**Authors:** Michelle F. Magee, Jacqueline E. Tamis-Holland, Jiang Lu, Vera A. Bittner, Maria Mori Brooks, Neuza Lopes, Alice K. Jacobs, BARI 2D Study Group

**Affiliations:** ^1^MedStar Health Research Institute at Washington Hospital Center and Georgetown University School of Medicine, MedStar Diabetes Institute, 100 Irving Street NW, No. 4114, Washington, DC 20010, USA; ^2^Mount Sinai Saint Luke's Hospital, New York, NY 10025, USA; ^3^University of Pittsburgh, Pittsburgh, PA 15261, USA; ^4^University of Alabama at Birmingham, Birmingham, AL 35294, USA; ^5^Heart Institute (InCor), 01238-000 São Paulo, SP, Brazil; ^6^Boston University Medical Center, Boston, MA 02118, USA

## Abstract

*Background.* Research has shown less aggressive treatment and poorer control of cardiovascular disease (CVD) risk factors in women than men. *Methods.* We analyzed sex differences in pharmacotherapy strategies and attainment of goals for hemoglobin A1c (HbA1c), blood pressure (BP), and low density lipoprotein cholesterol (LDL-C) in patients with type 2 diabetes and established coronary artery disease enrolled into the BARI 2D trial. *Results.* Similar numbers of drugs were prescribed in both women and men. Women were less frequent on metformin or sulfonylurea and more likely to take insulin and to be on higher doses of hydroxymethylglutaryl-CoA reductase inhibitors (statins) than men. After adjusting for baseline differences and treatment prescribed, women were less likely to achieve goals for HbA1c (OR = 0.71, 95% CI 0.57, 0.88) and LDL-C (OR = 0.64, 95% CI 0.53, 0.78). More antihypertensives were prescribed to women, and yet BP ≤ 130/80 mmHg did not differ by sex. *Conclusions.* Women entering the BARI 2D trial were as aggressively treated with drugs as men. Despite equivalent treatment, women less frequently met targets for HbA1c and LDL-C. Our findings suggest that there may be sex differences in response to drug therapies used to treat diabetes, hypertension, and hyperlipidemia.

## 1. Background

Control of blood glucose, blood pressure (BP), and low-density lipoprotein cholesterol (LDL-C) in patients with type 2 diabetes (DM) and cardiovascular disease (CVD) is key to achieve optimal outcomes [1]. Nationally, attainment of CVD prevention goals for patients with diabetes is suboptimal [2–6] and appears to be worse in women than in men [6–21]. This may be partially explained by a more adverse CVD risk profile in women and/or by differences in therapies given to women compared with men [6, 8, 11, 13, 15, 16, 22–25]. It is often difficult to determine how the dosing of these medications or the class of agents prescribed impact the differences in response to therapies that are seen by sex. Furthermore, less is known regarding whether there are also sex differences in response to drugs used for secondary CVD risk reduction. At present, there are no sex-based differences in guideline treatment recommendations for these three risk factors.

The bypass angioplasty revascularization investigation 2 diabetes (BARI 2D) trial was designed to evaluate outcomes in a cohort of patients with type 2 diabetes and known angiographically documented coronary artery disease (CAD), defined as one or more significant lesions deemed suitable for elective revascularization [26]. The BARI 2D baseline data set affords an opportunity to compare clinical characteristics and pharmacotherapy prescribing practices in a large cohort of middle-aged men and women with diabetes and CVD recruited 2001–2005. This paper compares the attainment of guideline recommended HbA1c, BP, and LDL-C benchmarks at study entry by sex and the relationship between number, type, and doses of drugs that were prescribed in women and men at study entry. We hypothesized that the approach to drug therapy would be similar in women and men who were enrolled in BARI 2D, and as such benchmark targets for HbA1c, BP, and LDL-C would also be similar by sex after adjusting for the number of relevant drugs prescribed.

## 2. Methods

BARI 2D (ClinicalTrials.gov Identifier: NCT00006305) is a multicenter, randomized NIH-funded trial designed to determine optimal treatment strategies for patients with DM and documented CAD suitable for elective revascularization. A detailed description of the study design and patient population has been previously reported [26]. Approval was obtained both from the University of Pittsburgh and from individual site institutional committees on human research. Subjects were recruited, consented, and randomized from 49 clinical sites in USA, Canada, Brazil, Mexico, the Czech Republic, and Austria between January 2001 and March 2005. Eligibility criteria included a diagnosis of DM and angiographically documented CAD not requiring immediate revascularization.

At the time of randomization, demographics, clinical history, physical exam, test results, and medications were collected. HbA1c and lipids were measured in a BARI 2D core laboratory and secondarily at point of care for clinical management decisions. Only those patients with quality baseline information were included in the present analysis. To classify level of control for study-designated treatment targets, measures of HbA1c, fasting LDL-C, and BP were collected. United States guideline recommendations for treatment goals for diabetes, hypertension, and cholesterol were set at <7% for HbA1c, <100 mg/dL for LDL-C, and ≤130/80 mm Hg for BP during the BARI 2D recruitment years [27] until 2004 when the LDL-C goal was tightened to allow consideration of <70 mg/dL [28]. Core laboratory derived HbA1c and LDL-C were available in 95% and 92% of patients, respectively. Missing core lab values were augmented by clinical site measures.

Therapeutic agents were categorized into antianginal/antihypertensive, antiplatelet/anticoagulant, antihyperlipidemic, and antidiabetes agents. Antidiabetes drugs were further subdivided into insulin providing (IP), insulin sensitizing (IS), and IP-IS neutral [29]. Each drug and its total daily dose at study entry were recorded. Diabetes agent and statin doses were further substratified to designate their being either within or above recommended starting dose(s) as stated in FDA approved prescribing information as of September 2007. The latter analysis was not performed for BP lowering drugs as these medications were not solely prescribed for BP control and we were not able to ascertain the indication (s) for which each BP agent was prescribed.

Statistical comparisons of proportions and means were made between sexes for demographic variables, clinical history, lab measures, and use of pharmacotherapeutic agents. For lipid lowering and oral diabetes agents, the proportion of patients whose clinical measures were at target was also compared by sex according to dose stratification. Chi-square tests and *t*-tests were performed as appropriate; *P* values less than 0.05 were considered statistically significant. In order to test sex differences in achieving treatment goals, outcomes of multiple logistic regression models for the defined targets of HbA1c and LDL-C BP were evaluated. Odds ratios of achieving treatment targets for women and men were calculated using logistic models adjusted for age, race ethnicity, education, physical activity, current cigarette smoking status, BMI, duration of diabetes, history of CAD prior to enrollment, hypertension, and number of relevant medications. All analyses were performed using SAS version 9.1.3 (Cary, NC).

## 3. Results

Among the 2368 patients enrolled, 2321 subjects had quality data and were included in the analysis. Among this group, there were 686 women, (mean age 62.9 years, 44.5% nonwhite) and 1635 men (mean age 62.2 years; 30.2% nonwhite). Demographic and clinical history characteristics are shown in Table 1. Women entering BARI 2D had a heavier burden of CVD risk factors than men, including higher BMI, longer duration of diabetes, higher prevalence of hypertension, a more sedentary lifestyle, and worse self-related health. Women less often had a history of cigarette smoking and were less likely than men to have had prior MI or CABG. Women had higher HbA1c and LDL-C levels and higher average BP than men.

Table 2 depicts the pharmacotherapeutic agents that were prescribed for women and men just prior to study entry. A similar percentage by sex was treated with most categories of agents, although significantly fewer women were taking metformin and sulfonylureas. Insulin and diuretics were being taken by more women than men. A similar number of women and men were taking some form of antiplatelet/anticoagulant; however, fewer women than men were taking aspirin.

The average number of drugs prescribed for each category of risk was determined. Within the category of antidiabetes agents, the number of drugs being taken did not differ between women and men (1.54 ± 0.81 versus 1.58 ± 0.88, *P* = 0.30). However, women were taking more antihypertensive drugs (2.36 ± 1.05 versus 2.17 ± 1.01, *P* < 0.001) and fewer lipid lowering drugs (0.84 ± 0.53 versus 0.91 ± 0.58, *P* = 0.004) than men. The average number of drugs being taken for the 4 risk categories assessed, including antiplatelet/anticoagulants, approached 7 agents and did not differ between women and men (6.64 ± 2.08 versus 6.57 ± 2.17, *P* = 0.43).

The average drug dose and the percentage of patients on titrated doses for statins and antidiabetes agents are shown in Table 3. Women were taking higher average doses of atorvastatin and pravastatin than men. For each antidiabetes drug, average doses did not differ and the percentages on titrated doses were similar by sex, except for the percentage on >0.4 units/kg/day of insulin which was being taken by more women. There were no significant differences in the percent of women and men taking statin dose above the recommended starting dose(s) per FDA approved prescribing information.

An analysis by sex of the percent of subjects who met the prespecified clinical targets for HbA1c, BP, LDL-C, and BP is detailed in Table 4. Significantly, fewer women were at goal for both HbA1c and LDL-C than men. There was no difference by sex in the percent at target for BP. After adjustment for covariates, including clinical variables as well as number of relevant agents prescribed, the odds of being at target for HbA1c and LDL-C remained significantly lower for women than for men. The odds of being at target for BP remained similar.

## 4. Discussion

Control of CVD risk factors substantially improves outcome among high-risk patients with DM [1]. Based on this information, clinical guidelines specific to individuals with diabetes that were in effect at the time of BARI 2D recruitment specified benchmark targets for control of HbA1c, LDL-C, and BP. The BARI 2D baseline data analysis allows comparison of physician prescribing practices, intensity of drug therapy prescribed, and degree of attainment of standards of care for HbA1c, LDL-C, and BP in women and men with DM and established CAD across a diversity of physician practice settings. The findings of these baseline data suggest that women enrolled in the BARI 2D trial were as intensively treated with drugs for DM and CVD prevention as men at study entry, with the exception of aspirin which was taken by fewer women than men. Despite equivalence in prescribing practices, women met benchmark targets for HbA1c and LDL-C less often than men. The adjusted odds ratio was in the same direction and of similar magnitude for HbA1c and LDL-C, compared with the unadjusted odds. This demonstrates a robust relationship between sex and achievement of targets. Our findings are consistent with some prior reports that demonstrated that women are less likely than men to achieve control of HbA1c [6, 8, 11, 17, 18] and LDL-C [6–11, 17–21]. A few studies have reported on the likelihood of achieving guideline targets after adjusting for the type of drugs prescribed [11, 12, 30–32]. In the recent report by Rossi et al., of a cohort of Italian diabetes clinic patients, inequalities in attainment of benchmarks were observed with women being more likely than men to have A1C >9.0% in spite of insulin treatment and to have LDL >130 mg/dL in spite of lipid lowering treatment [31], as was the case in the BARI2D cohort. Both the BARI2D and the Italian cohorts demonstrated medical undertreatment of women with aspirin and in the latter undertreatment with ACE-inhibitors was also observed among females. In contrast to these findings, among a Swedish cohort of adults with chest pain referred for a first time diagnostic elective coronary angiography from 2006 to 2008, it was shown that female sex was independently associated with underutilization of guideline recommended therapy. The Swedish data revealed subsequent equivalent use of ACE-inhibitors, beta-blockers, aspirin, and statins among women and men after angiographic diagnosis of obstructive CAD was made [32].

Our detailed analysis of pharmacotherapeutic agents used to control HbA1c, LDL-C, and BP among patients entering the BARI2D study demonstrates that, despite a similar intensity of medications used to control CVD risk factors, women were still less likely to achieve target goals for HbA1c and LDL-C than men. Women enrolled in the BARI 2D study had a less favorable risk profile at baseline compared with men, including greater age, longer duration of DM, higher BMI, a higher prevalence of hypertension, a more sedentary lifestyle, and a lower level of education. Although these variables might affect the ability to control HbA1c, LDL-C, and BP, differences were still noted among women and men even after adjustment for these variables, suggesting that alternate factors are likely at play in this sex gap. Various other explanations for the observation that women are less likely than men to reach treatment goals for HbA1c, LDL-C, and BP have been put forward, including biologic factors, medication adherence, and possible differences in the quality of health care delivery by sex.

Women have been shown to receive less aggressive therapies to treat or prevent CVD than men [6, 8, 11, 13, 15, 16, 22–25, 31–33]. In the current report, however, we performed a detailed analysis of number and intensity of medications prescribed for women and men and found very similar dosing of medications used to treat CVD risk factors. If anything, the intensity of therapy was greater in women than men. More women than men were treated with insulin in keeping with their longer duration of diabetes and higher HbA1c levels. Insulin doses (units/kg body weight/day) were also higher in women. The findings that more women than men in BARI 2D were treated with insulin and that women were taking a higher number of units of insulin/kg of body weight daily when compared to men suggest the possibility of a greater degree of insulin resistance among the women. This possibility is also supported by the presence of a higher BMI and a more sedentary lifestyle among the women subjects. Statins were prescribed to a similar percentage of women and men, and a similar number by sex were on a “titrated” dose of statin. The average dose of statins tended to be higher in women. Although fewer women than men were treated with fibrate therapy, the total number of drugs used to treat hypercholesterolemia was similar by sex. On average more total antihypertensive drugs were prescribed to women than men. A similar percentage of women and men were taking an ACE-inhibitor or ARB and/or beta-blockers and more women than men were taking diuretics. The only CVD prevention agent which was prescribed less frequently among BARI2D women was aspirin. Given these findings, we do not feel that the differences reported were a result of sex differences in prescribing practices.

It is possible that there are inherent biological differences by sex in the response to the pharmacotherapeutic agents used to treat CVD. For example, studies have shown sex differences in the biologic and clinical response to antiplatelet drugs [34, 35], while other studies have suggested differences in the time to achieve adequate control of LDL-C as a function of race and sex [30]. Differences in response to therapies may relate to sex differences in enzymatic activities, glomerular filtration, levels of endogenous hormones, body surface area, and proportion of body fat. It is possible that these biologic differences in women and men impact the efficacy of the drugs used to treat DM, high cholesterol, and hypertension. Previous reports have demonstrated poorer compliance with medications and lifestyle interventions in women than men [7, 20, 36] and an association of adherence to medications and achievement of target goals [7]. Compliance with medications can be influenced by the cost of the medication, the patient's underlying condition, the frequency of follow-up visits, the use of mail order pharmacies, and sociodemographic variables [20]. Information regarding adherence to therapy prescribed and dietary and lifestyle practices at study entry in BARI 2D was not recorded. We did, however, show that women enrolled in BARI 2D were older than men, had a higher BMI, led more sedentary lifestyles, and had poorer education; all of these factors may directly impact adherence. Our multivariate model attempted to adjust for these variables; however, even after adjustment, sex differences in achievement of goals persisted.

It is also possible that the differences in the outcomes reported by sex relate to higher pretreatment levels of HbA1c, LDL-C, and BP. Some studies have demonstrated that the ability to adequately lower LDL-C is directly correlated with starting LDL-C values [37]. Since we did not have information regarding the “pretreatment” indices for HbA1c, LDL-C, and BP, we cannot determine whether these parameters had any effect on achievement of benchmarks for these targets.

## 5. Limitations

Baseline data presented in this analysis were obtained by each subject's self-report. Therefore, information on drugs previously prescribed, their side effects, and information on subject adherence with the prescribed therapy were not available. Each of these variables could potentially impact sex differences in target attainment and limits our ability to determine whether the differential attainment of targets between the sexes is due to adherence factors or actual response to therapy. In addition, the recruitment of subjects from academic medical centers may bias the data, limiting the ability to extrapolate findings to community practices.

## 6. Conclusions

Women enrolling in BARI 2D were being treated as intensively with diabetes, lipid lowering, and blood pressure drugs as men. Despite equivalence of therapies prescribed, including number of agents prescribed and an apparently equivalent degree of drug dose titration, women less frequently met targets for HbA1c and LDL-C than men. These findings suggest that sex differences in attaining clinical targets cannot be explained solely by sex bias in drug prescribing practices. Other variables such as differences in medication adherence or differences in therapeutic responses to agents used for secondary CVD prevention among women compared to men must be considered. As we strive to decrease the percentage of both women and men with type 2 diabetes who die from CVD, further studies are needed to investigate sex-specific factors that may impact targeted management of risk factors for CVD.

## Figures and Tables

**Table 1:** BARI 2D demographics and clinical history by sex.

Demographic/clinical history	Female (*N* = 686)	Male (*N* = 1635)	*P* value
Age at entry (years), mean ± SD	62.9 ± 9.3	62.2 ± 8.7	0.08
Race ethnicity			***<0.001***
White, non-Hispanic	55.5%	69.8%	
Black or African-American, non-Hispanic	27.7%	12.4%	
Hispanic	13.0%	12.5%	
Asian and others	3.8%	5.3%	
Region and country			***<0.001***
United States and Canada	74.9%	79.1%	
México and Brazil	22.1%	17.7%	
Czech Republic& Austria	3.1%	3.3%	
Education level			***<0.001***
<High school	46.1%	33.0%	
≥High school	53.9%	67.0%	
BMI (kg/m^2^), mean ± SD	32.9 ± 6.9	31.3 ± 5.4	***<0.001***
Diabetes duration (years), mean ± SD	12.2 ± 9.6	9.7 ± 8.1	***<0.001***
History of hypertension	87.2%	80.4%	***<0.001***
History of hypercholesterolemia	82.9%	81.4%	0.4
History of MI	27.9%	33.6%	***0.007***
History of CHF	7.8%	6.0%	0.1
CABG prior to randomization	4.5%	7.2%	***0.02***
PCI prior to randomization	20.1%	19.4%	0.7
Cerebrovascular Accident	11.0%	9.3%	0.2
Physical Activity			***<0.001***
Sedentary	28.2%	19.3%	
Mild to moderate	69.6%	77.4%	
Strenuous	2.2%	3.3%	
Cigarette smoking			***<0.001***
Never smoked	51.9%	25.4%	
Current smoker	9.8%	13.4%	
Self-rated health			***<0.001***
Excellent to good	46.8%	56.4%	
Fair to poor	53.3%	43.6%	
Mean HbA1C (%)	8.0 ± 1.7	7.5 ± 1.6	***<0.001***
Mean blood pressure (mm Hg)			
Systolic (mm Hg)	134.8 ± 22.6	130.4 ± 18.7	***<0.001***
Diastolic (mm Hg)	73.8 ± 12.5	74.9 ± 10.6	***0.04***
Mean LDL cholesterol (mg/dL)	102.9 ± 34.8	93.5 ± 32.2	***<0.001***

MI = myocardial infarction; CHF = congestive heart failure.

**Table 2:** Pharmacotherapeutic agents by target category and by class.

Pharmacotherapeutic agent	Total (*N* = 2321)	Female (*N* = 686)	Male (*N* = 1635)	*P* value
*Antidiabetes agents *				
Any diabetes drug	91.4%	92.1%	91.1%	0.44
Insulin sensitizing	60.9%	57.7%	62.3%	***0.04***
Metformin	54.1%	50.2%	55.7%	***0.02***
TZD	18.8%	16.4%	19.8%	0.06
Insulin Providing	75.6%	77.8%	74.7%	0.11
Sulfonylurea	53.6%	47.7%	56.0%	***<0.001***
Meglitinide	0.7%	1.0%	0.6%	0.29
Insulin	27.8%	36.3%	24.3%	***<0.001***
*Lipid lowering agents *				
Any lipid drug	79.1%	77.3%	79.9%	0.16
Statin	74.7%	73.0%	75.4%	0.22
Fibrate	8.6%	6.3%	9.6%	***0.01***
Niacin	2.2%	1.5%	2.4%	0.14
*Blood pressure agents *				
Any blood pressure drug	95.8%	95.8%	95.8%	1.00
ACE or ARB	77.1%	75.6%	77.7%	0.27
Beta-blocker	72.9%	74.3%	72.3%	0.32
Calcium channel blocker	31.4%	33.9%	30.4%	0.10
Diuretic	38.7%	49.9%	34.0%	***<0.001***
*Antiplatelet/anticoagulants *				
Any antiplatelet/anticoagulant	91.9%	90.5%	92.5%	0.10
Aspirin	88.0%	85.7%	89.0%	***0.03***
Ticlopidine/clopidogrel	18.0%	18.8%	17.6%	0.49

ACE = angiotensin converting enzyme inhibitor; ARB = angiotensin receptor blocker.

**Table 3:** Average daily drug dose and percentage on titrated doses for diabetes agents and statins by sex.

Agent	*N*		Average daily dose (mg)			% on titrated dose			
	Female	Male	Female	Male	*P* value	Threshold	Female	Male	*P* value
Taking diabetes drug	**622**	**1465**	**—**	**—**	**—**	Diabetes drugs	**72%**	**73%**	**0.63**
*Any insulin sensitizing *	**394**	**1015**	**—**	**—**	**—**	IS	**58%**	**62%**	**0.27**
TZD	111	322	—	—	—	TZD	46%	42%	0.46
Pioglitazone	61	139	32.5	31.3	0.49	>30 mg	36%	32%	0.54
Rosiglitazone	50	183	6.7	6.0	0.12	>4 mg	58%	50%	0.30
Metformin	344	907	1517.5	1526.2	0.83	>1000 mg	58%	61%	0.31
*Any insulin providing *	**515**	**1162**	**—**	**—**	**—**	IP	**71%**	**69%**	**0.34**
Sulfonylurea	316	864	—	—	—	SU	63%	64%	0.66
Glyburide	192	540	18.0	13.5	0.30	>5 mg	75%	73%	0.58
Glipizide	87	236	17.6	14.0	0.31	>10 mg	31%	44%	***0.03***
Glimepiride	37	88	5.1	4.3	0.17	>2 mg	76%	66%	0.28
Insulin	249	397	0.7 unit/kg	0.6 unit/kg	0.17	>0.4 unit/kg	75%	68%	***0.04***
*Taking statin *	**483**	**1186**	**—**	**—**	**—**	Statins	**46%**	**46%**	**0.96**
Atorvastatin	181	443	29.4	25.0	***0.01***	>10 mg	66%	62%	0.36
Lovastatin	16	42	28.8	35.5	0.18	>20 mg	38%	64%	0.07
Pravastatin	41	92	32.9	24.8	***0.002***	>40 mg	5%	1%	0.17
Simvastatin	245	609	28.8	29.8	0.53	>20 mg	39%	41%	0.71

IS = insulin sensitizing; IP = insulin providing diabetes drugs; SU = sulfonylurea.

**Table 4:** Achievement of clinical targets for HbA1c, blood pressure, and LDL-cholesterol at baseline in BARI 2D by sex.

Clinical target	% at clinical target			Odds ratio (95% CI)^†^	
	Female (*N* = 686)	Male (*N* = 1635)	*P* value	Unadjusted female versus male	Adjusted^†^ female versus male
HbA1c < 7%	31.9%	42.3%	***<0.001***	0.64 (0.53, 0.77)	0.71 (0.57, 0.88)
Blood pressure ≤ 130/80 mm Hg	45.0%	48.6%	0.12	0.87 (0.72, 1.04)	1.11 (0.92, 1.35)
LDL < 100 mg/dL	49.8%	63.1%	***<0.001***	0.58 (0.48, 0.69)	0.64 (0.53, 0.78)
Achieved all 3 target goals	10.9%	15.8%	***0.002***	0.65 (0.49, 0.86)	0.78 (0.58, 1.04)

^†^All clinical targets were adjusted for the following common covariates: sex, age, race ethnicity, education, physical activity, cigarette smoking, duration of diabetes, and BMI. In addition, HbA1c was adjusted for a number of diabetes agents; lipids targets were adjusted for a number of lipids agents and CABG or PCI prior to randomization; blood pressure target was adjusted for a number of antihypertensive agents, CABG or PCI prior to randomization, and history of hypertension. The attainment on all 3 targets was controlled for a number of total drugs, CABG or PCI prior to randomization, and history of hypertension.
